# Optimizing nuclear-renewable hybrid energy systems for cost efficiency based on energy security concerns in Puerto-Rico

**DOI:** 10.1038/s41598-026-46862-7

**Published:** 2026-04-02

**Authors:** Rita Appiah, Diego Aguilar, Jhon Quiñones, Luciano Castillo

**Affiliations:** 1 School of Nuclear Engineering, Purdue University, West Lafayette, 47907 USA; 2 School of Electrical and Computer Engineering, Purdue University, West Lafayette, 47907 USA; 3 School of Mechanical Engineering, Purdue University, West Lafayette, 47907 USA; 4 Faculty of Electrical Engineering, Mathematics and Computer Science, Delft University of Technology, 2628 XE Delft, The Netherlands

**Keywords:** Levelized cost of energy, Buckingham $\pi$-theorem, Optimization, Hybrid renewable energy system, Energy security, Energy science and technology, Engineering, Mathematics and computing

## Abstract

This study develops an optimized scientific framework to identify least-cost energy mixes while enabling scale-invariant energy security assessment for Puerto Rico’s clean-energy transition. A nonlinear programming model is formulated to minimize total energy cost, and a Gaussian Process Regression (GPR) surrogate with explainability is employed to identify key cost drivers and quantify techno-economic uncertainty. To address the complexity of hybrid energy systems, fifteen relevant Nuclear–Renewable Hybrid Energy System (N-RHES) features are systematically aggregated into six energy security variables representing system capacity, storage, renewable penetration, and demand characteristics. Using these variables, a dimensional-scaling framework based on the Buckingham $\pi$-theorem is developed to construct three dimensionless $\pi$-groups corresponding to Reliability, Resilience, and Renewability (3R). These metrics transform system-specific optimization outputs into transferable, scale-invariant engineering performance indicators suitable for comparing islanded energy systems of different sizes. The GPR surrogate provides posterior mean predictions $\mu (\textbf{x}^*)$ and predictive variance $\sigma ^2(\textbf{x}^*)$ to characterize uncertainty in Levelized Cost of Energy (LCOE) and energy security metrics. SHapley Additive exPlanations (SHAP) analysis indicates that nuclear capacity reduces LCOE by 1.4 ¢/kWh, whereas wind increases cost by 0.9 ¢/kWh in high-penetration scenarios. Under techno-economic uncertainty, the predicted LCOE is $9.6\pm 2.3$ ¢/kWh, with the optimal nuclear–hybrid solution achieving 9.6 ¢/kWh while remaining below the 11.0 ¢/kWh policy constraint. Five hybrid configurations combining wind, solar PV, geothermal generation, battery storage, and hydrogen fuel-cell systems are analyzed, with selected cases integrating Small Modular Reactor (SMR) base-load supply. Optimization identifies three recommended configurations, with an SMR–renewables hybrid emerging as the least-cost solution. Configuration 5 achieves an LCOE of 10.0 ¢/kWh, delivers 70% renewable contribution, and reduces total energy cost by 18% relative to fossil-dominant mixes. By integrating techno-economic optimization with $\pi$-based dimensional scaling, the proposed framework provides physically interpretable and transferable energy security metrics applicable to heterogeneous hybrid energy systems and hurricane-exposed island grids.

## Introduction

Puerto Rico (PR) faces significant challenges regarding sustainable and reliable energy supply due to its geographic isolation, aging infrastructure, and increasing susceptibility to extreme weather events^1^. The island’s heavy dependence on imported fossil fuels has resulted in high energy costs and vulnerability to supply interruptions. With the global acceleration of energy transitions, there is an urgent need for Puerto Rico to embrace innovative alternatives that prioritize cost efficiency, resilience, and sustainability.

Hybrid Renewable Energy Systems (HRES), combining resources such as wind, solar photovoltaic (PV), hydrogen, and low-carbon base-load options like Small Modular Reactors (SMRs), have emerged as promising solutions for improving the resilience and sustainability of islanded energy systems. However, these hybrid systems involve numerous interacting technical, operational, and economic variables, making the assessment of key energy security metrics and overall system feasibility challenging, particularly when only limited system-level data are available. Existing studies often rely on absolute indicators such as installed capacity, storage size, and LCOE, which remain dependent on system scale and local units and therefore limit cross-system comparability^2^. To address this gap, this study develops a scalable optimization framework using Puerto Rico as a representative case and introduces a scientifically grounded approach based on the Buckingham–$\pi$ theorem to derive dimensionless engineering performance metrics that characterize energy security. These metrics scale key system variables and provide a scale-independent basis that complements existing optimization approaches for evaluating Nuclear–Renewable Hybrid Energy Systems (N-RHES) across islanded grids of varying sizes, offering transferable insights for other Caribbean islands with similar grid configurations.

The remainder of this paper is structured as follows. The next section presents a literature review on existing metrics for evaluating energy feasibility, the specific challenges of island energy systems, and an overview of previous studies focused on Puerto Rico. Following this, we detail our optimization model, the derivation of our proposed energy security metrics, and the implementation of analysis tools such as Gaussian Process Regression. We then present results analyzing how different energy configurations impact these metrics. The document concludes with a summary of the main findings and significance of the study.

## Literature survey

### Sustainability of renewable systems

The global effort to achieve net-zero emissions by 2050 and to limit global warming to $1.5\,^\circ \textrm{C}$ has become an urgent priority, placing a critical responsibility on the power sector to lead a rapid and coordinated energy transition^3^. Meeting these targets requires integrating diverse clean energy sources (e.g., renewables, nuclear, and hydrogen) alongside cutting-edge technologies like small modular reactors (SMRs), Hybrid Renewable Energy Systems (HRES), and Artificial Intelligence (AI) for optimal modeling and forecasting. Optimizing these configurations is essential for ensuring grid reliability under variable conditions and supporting an increasingly complex energy matrix.

Evaluating the viability of a particular energy mix has become the subject of various studies. The sustainability of HRES may be evaluated through environmental, economic, technical, social, and institutional metrics^4^, which are typically computed with techniques like Life Cycle Assessment (LCA) or Principal Component Analysis (PCA). A thermodynamic perspective is also found in the literature, where concepts like Energy Return on Investment (EROI) or Energy Payback Time (EPBT) are used to assess energy efficiency over the lifecycle^5^. These physical indicators complement financial metrics such as the Levelized Cost of Energy (LCOE), which describes, in $/MWh, the cost of operating a power plant over its expected lifetime while considering all associated costs. In the case of renewables, it has been found that the LCOE of utility-scale solar PV and onshore wind have dropped by 89% and 69%, respectively, between 2010 and 2022, making renewables increasingly competitive against conventional fossil fuels^6^. Finally, the technical reliability and resilience of a renewable system must be evaluated. Here, the focus shifts to how the grid performs under real-life conditions such as power outages, dynamic demand, energy storage, and increasingly complex technologies. Some classical metrics include Loss of Load Expectation (LOLE) and Expected Energy Not Supplied (EENS), while recent studies propose new concepts such as availability and resiliency indices to better capture the contribution of renewables to grid stability^7^.

### Challenges in island energy systems

Island energy systems typically face a distinct set of challenges, including high costs and price volatility driven by reliance on imported fuels, the absence of mainland grid interconnections, and significant seasonal variability in both demand and renewable resource availability. Despite technological advancements, energy production in many remote areas and small islands remains dominated by fossil fuels due to these unique constraints. However, global efforts towards decarbonization have driven research into maximizing the exploitation of local renewable sources. For instance, a case study conducted in the Aeolian Islands demonstrated that optimizing a mix of solar, wind, and marine energy could significantly mitigate these dependencies. By extending the LCOE metric to evaluate the collective performance of the combined sources rather than individual technologies, the study identified a configuration capable of meeting 50% of the summer electrical load. The results indicated that a combination of wind (45.47%), solar (10.2%), and innovative sea wave technologies (3.04%) could reduce the share of diesel generation to approximately 41.29%^8^.

Beyond static cost metrics, recent literature has explored integrated methodologies to assess energy security under uncertainty, such as using the Open Source Energy Modeling System (OSeMOSYS) to simulate stochastic disturbances. This approach introduces an “energy security coefficient” to quantify trade-offs between price stability and unsupplied energy, identifying technology shares that maintain security during shocks^9^. Simultaneous optimization of supply and demand has also emerged, with novel models, typically formulated using Mixed-Integer Linear Programming (MILP), simulating energy flows at an hourly resolution to evaluate complex design spaces^10^. By integrating demand-side management with generation, these frameworks optimize conflicting objectives like life cycle cost, carbon emissions, and system efficiency. At the building scale, heuristic methods such as genetic algorithms have been coupled with dynamic simulation engines to minimize primary energy demand and investment costs, offering a global search alternative to gradient-basedNon-Linear Programming (NLP) when addressing non-convex regulatory constraints^11^. There are also models that consider strict policy targets by analyzing hourly data to minimize the overall required power generating capacity. These approaches identify “near-optimal” domains of renewable combinations, offering policymakers flexibility in achieving penetration targets while maintaining load-following capabilities^12^.

However, effective transition strategies require not only optimizing electricity generation but also developing stepwise pathways that account for neglected sectors and consider potential interconnections with neighboring regions. For example, while solar PV and wind are the predominant technologies, their deployment is frequently hampered by strict area limitations, a constraint particularly acute in island nations. Additionally, current design approaches often fail to adopt a multi-sectoral perspective, leaving significant gaps in addressing demands from maritime transport, aviation, and complex water systems. Recent studies also emphasize the role of transport electrification as a flexible component of integrated energy systems, where coordinated electric vehicle charging infrastructure can provide distributed demand-side flexibility and support renewable integration within transmission and distribution networks^13^. Capturing the complexities of non-interconnected island systems requires advanced modeling tools beyond the capabilities of standard planning software. Recent methodological developments have sought to address this by explicitly linking energy and economy models to capture sectoral interactions, including heating, cooling, and mobility^14^. Therefore, while islands contribute relatively little to global climate targets, their unique geographical constraints position them as potential front runners in the clean energy transition. Puerto Rico is a prime case study of a territory facing these adverse conditions, grappling with geographic isolation, heavy dependence on imported fossil fuels, and high electricity consumption^15^. This reliance increases exposure to price volatility, while extreme weather remains the leading cause of large-scale power failures^16^. Hurricane María in 2017 exposed the fragility of the centralized grid, destroying 80 % of transmission lines and causing the longest blackout in U.S. history with economic losses exceeding $90 billion^17,18^.

Beyond these physical and economic risks, the integration of renewables is limited by inadequate land space, grid instability, and the need for complex management systems for intermittent sources^19^. Regulatory constraints and insufficient funding further delay the energy transition. Consequently, the literature emphasizes that modernizing the infrastructure toward resilient, decentralized architectures is urgent. Integrating renewables, storage, and modular nuclear systems has been identified as a critical pathway to strengthen reliability and reduce fossil fuel dependence, necessitating robust metrics to evaluate these configurations against extreme events.

### Summary of previous studies of Puerto Rico’s energy transition

Numerous studies have assessed Puerto Rico’s renewable energy potential, primarily emphasizing solar, wind, and distributed generation systems aimed at reducing reliance on fossil fuel imports^20^. These analyses also underscore key technical challenges, including resource intermittency, storage limitations, and infrastructure constraints^21^.

**Fig. 1:**
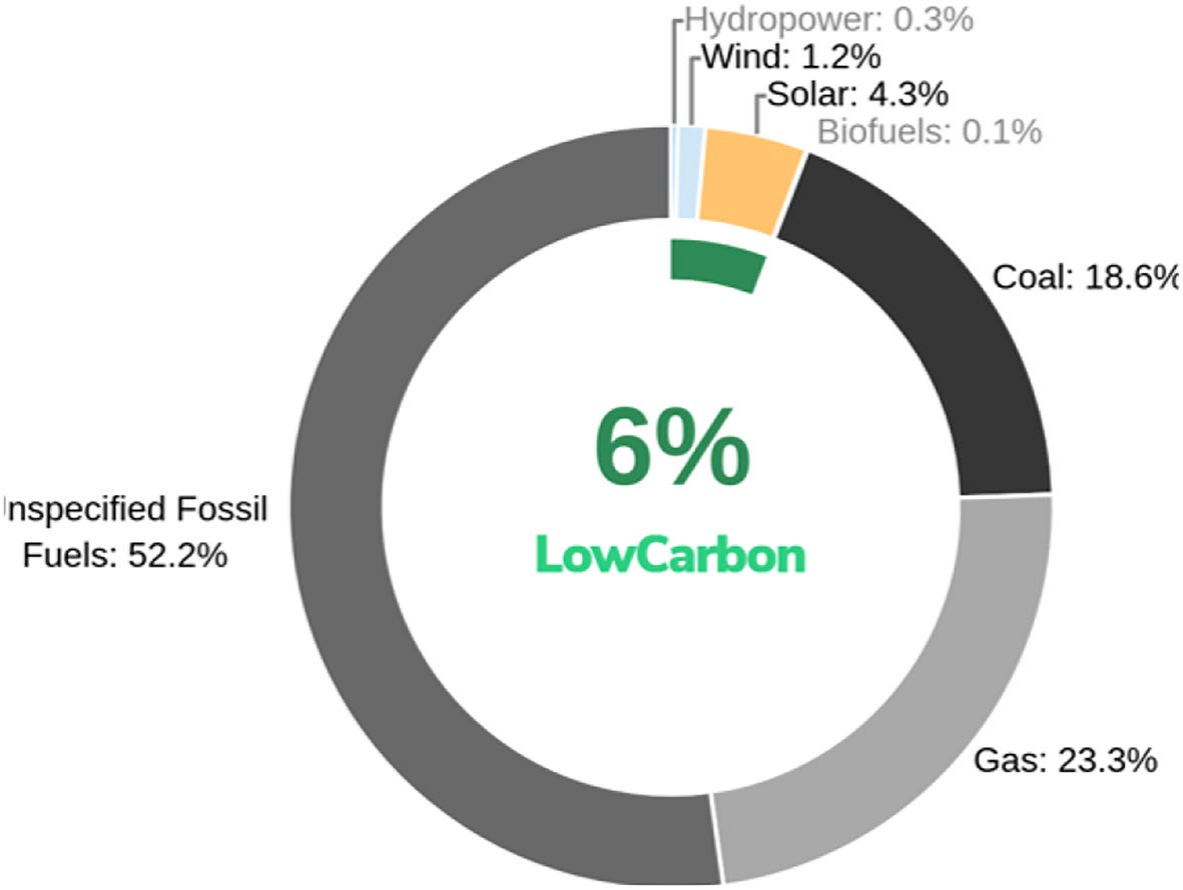
Puerto Rico electricity generation mix in 2024 highlighting fossil fuel dominance with limited low-carbon contribution^22^.

Figure 1 illustrates Puerto Rico’s current electricity generation mix, which remains heavily dominated by fossil fuels, highlighting the limited penetration of low-carbon energy sources and emphasizing the need for resilient and diversified energy transition strategies.Furthermore, the study conducted by^23^ explored distributed renewable systems, but their approach emphasized the need for a more robust optimization approach that incorporates economic and performance-centered resilience analysis under extreme weather events.

The 100 percent renewable electricity target for Puerto Rico projected by the year 2050 was the focus of the most recent PR100 study conducted by NREL^24^. According to the study conducted by NREL, renewable electricity has the potential to improve the economy, however, flexible resources, diverse combinations of renewable generation, investments in grid modernization, and battery energy storage systems are essential.

Complementary feasibility assessments have also explored the potential role of advanced nuclear technologies, including Small Modular Reactors (SMRs) and micro-reactors, as resilient low-carbon generation options for Puerto Rico’s islanded grid, highlighting their potential contribution to energy security, grid stability, and disaster resilience^25^.

The proposed framework presented in this document addresses these gaps by employing PR-specific technologies to optimize the supply with a flexible, reliable and cost-effective hybrid system.

### Novel contributions of this study

Despite the extensive literature on renewable energy optimization, existing studies often treat economic metrics (e.g., LCOE) and technical reliability (e.g., LOLE) in isolation, or rely on computationally expensive simulations that lack interpretability for policymakers. Moreover, while islanded systems like Puerto Rico have been studied, few frameworks explicitly quantify the trade-offs between resilience and cost using dimensionless metrics that allow for cross-regional comparison. There is a distinct need for a methodology that not only optimizes component sizing but also provides explainable insights into how specific energy mixes, including emerging options like SMRs, perform under uncertainty.

To address these limitations, this study introduces a framework that integrates three key innovations:

1. A dimensionless 3R metric for energy security (Reliability, Resilience, Renewability) derived from the Buckingham $\pi$-theorem, enabling standardized assessment across different island grids.
2. An adaptable Nonlinear Programming (NLP) model that determines the optimal sizing of hybrid power plant configurations, explicitly accounting for non-linear economies of scale often simplified in linear models.
3. The application of Explainable AI, specifically Gaussian Process Regression (GPR) coupled with SHapley Additive exPlanations (SHAP), to quantify uncertainty and interpret the complex trade-offs in design decisions.

These contributions enable scalable, interpretable, and uncertainty-aware energy planning tailored to the unique resilience needs of Puerto Rico and similar islanded regions.

Table 1 compares this study with prior energy system models in terms of resilience, uncertainty, and interpretability. This work uniquely integrates a dimensionless $\pi$-group resilience metric, predictive uncertainty using Gaussian Process variance, and explainable Artificial Intelligence (XAI). While prior studies have examined individual aspects of hybrid energy system modeling, few combine techno-economic optimization, resilience assessment, and interpretable analytics within a unified framework. This study introduces a transferable methodology that couples nonlinear cost optimization with dimensionless Reliability–Resilience–Renewability (3R) metrics derived from the Buckingham $\pi$-theorem. The resulting $\pi$-parameters provide a linearly scalable scientific framework for evaluating the engineering performance of hybrid energy systems across deployment scales, even with limited resource or infrastructure data. Applied to Puerto Rico as a representative island grid, the framework offers interpretable and uncertainty-aware decision support for assessing project feasibility and informing energy planning and policy decisions.

**Table 1:** Full comparative table of this study versus existing hybrid energy modeling efforts.

References	Model	Objective	Resilience metric	Uncertainty treatment	XAI	Energy sources
^24^	Scenario planning + stakeholder input	100% renewable energy for PR by 2050	Scenario-driven outage duration modeling	Implicit (multi-scenario)	✗	Solar PV, wind, hydro, battery
^23^	Grid investment + resilience modeling	Enhance reliability using DERs	Event-based infrastructure resilience	Scenario flexibility	✗	DERs, microgrids
^21^	Policy review of energy transition	Identify transition pathways in Caribbean	Narrative-based resilience (no quant. model)	qualitative	✗	Solar, wind, storage, grid
^26^	Techno-economic hybrid simulation	Off-grid clean supply	Storage autonomy and backup config	Resource / load variation	✗	Solar, wind, diesel, battery
^27^	HOMER microgrid design	Rural microgrid resilience	Blackout duration and autonomy-based resilience	Scenario-based variation	✗	Solar, diesel, battery, wind
^28^	GEKKO grid optimization	LCOE reduction and grid balancing	Grid flexibility under outage events (implicit)	Load + line variation	✗	Solar, wind, grid, battery
^25^	SMR feasibility and deployment model	Evaluate SMR potential in PR grid	base-load firming and grid reliability (qualitative)	Policy, financial, scenario variation	✗	nuclear SMRs, limited RE
This study	NLP optimization + GPR surrogate + $\pi$-group metrics	LCOE minimization + 3R energy security for PR by 2050	$\pi$-group metric scaling storage vs. critical demand	Predictive variance $\sigma ^2(x^*)$, SHAP sensitivity	✓	Solar, wind, nuclear SMR, geothermal, H$\phantom{0}_2$/FC, battery

## Methods

This section outlines the multi-step modeling approach that integrates a techno-economic assessment of the optimal hybrid energy mix for PR, followed by a dimensional analysis of energy security metrics. A Non-Linear Programming (NLP) model was employed to minimize the total cost across a variety of renewable and dispatchable energy sources. Using the Buckingham $\pi$-theorem, the 3R Metrics were developed as dimensionless performance indicators to improve generalization. These $\pi$ groups make it possible to compare hybrid energy systems consistently across a range of geographical and technical contexts. The NLP optimization framework was implemented in Python 3.10 using SciPy’s SLSQP optimizer for nonlinear programming. Model parameters include techno-economic inputs such as capital and operating costs (CAPEX, OPEX), discount rate, and efficiency, which were non-dimensionalized using Buckingham $\pi$-group scaling to define the 3R energy security metrics: Reliability, Resilience, and Renewability. Parameter definitions and ranges are summarized in Table 2.

Additionally, a mathematical formula that relates the operational characteristics of each energy source to the annual and specific water use (gal/MWh) was used to estimate the water consumption for each hybrid configuration. Metrics for the Return on Investment (ROI) are also presented.

This formulation is supported by a Gaussian Process Regression (GPR) model also implemented in Python 3.10 using Scikit-learn library for Gaussian Process Regression (GPR) modeling, and the SHAP library to enhance the interpretability and analysis of the techno-economic parameters assessed. GPR is a probabilistic approximation of the optimization landscape that allows for sensitivity analysis and provides insight into the ways in which input parameters affect important outputs. The primary technical criteria guiding the optimization are: (i) a capacity adequacy constraint ensuring total installed capacity meets or exceeds peak demand in (6), (ii) an LCOE constraint enforcing cost-effectiveness of the hybrid system in (7), and (iii) secondary evaluation via dimensionless energy security metrics—Reliability (R_1_), Resilience (R_2_), and Renewability (R_3_), as defined in Table 4.

**Fig. 2:**
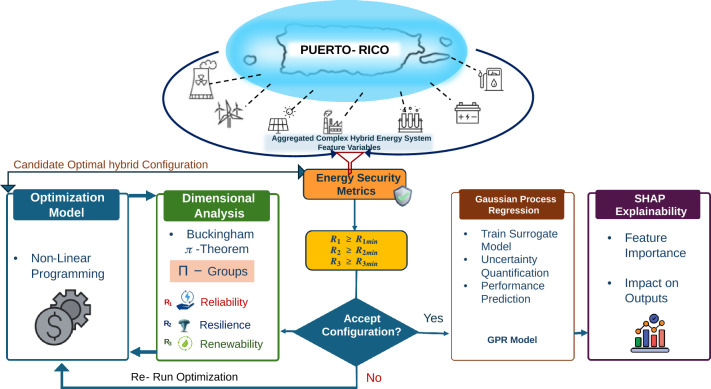
Implemented N-RHES Optimization workflow framework for the Puerto-Rico case study.

The modeling framework, data sources, and dimensional analysis methodology employed in the study are described in detail in the following sections. The computational workflow used in implementing this methodology is illustrated in Fig.  2 and summarized procedurally in Algorithm 1.

### Optimization model formulation

The optimal energy mix for a specific region is determined by a formulation that integrates local demand data, generation rates, and predefined economic targets. The set of energy sources considered in the analysis, denoted by $\mathcal {S}$, encompasses a range of conventional and renewable options:

1 $$\begin{aligned} \mathcal {S} = \{ \text {fos, gas, nuc, hfc, wnd, spv, sth, hyd, geo} \}\, \end{aligned}$$

where the elements represent fossil fuels (fos), natural gas (gas), nuclear (nuc), hydrogen fuel-cell storage (hfc), wind (wnd), solar photovoltaic (spv), solar thermal (sth), hydropower (hyd), and geothermal (geo).

In the optimization formulation, the hydrogen fuel-cell subsystem provides long-duration energy storage through hydrogen production and reconversion to electricity. For notational simplicity in the decision variables and system equations, this storage functionality is represented by the generalized bulk energy storage variable *bes*. Thus, the decision variable $x_{bes}$ used in the optimization corresponds to the hydrogen storage–fuel cell subsystem. We may also define a subset for the renewable sources: $\mathcal {R}=\{ \text {wnd}, \text {spv}, \text {sth}, \text {hyd}, \text {geo} \}$.

#### Decision variables

The primary decision variables for the optimization are the installed capacities for each energy source. These are represented by a vector $\textbf{x} \in \mathbb {R}^{N_s}$, where $N_s$ is the total number of available energy sources in the set $\mathcal {S}$. Each element $x_s$ in the vector corresponds to the plant size in kilowatts (kW) for a given source $s \in \mathcal {S}$. The decision vector $\textbf{x}$ is explicitly defined as:

2 $$\begin{aligned} \textbf{x} = \begin{bmatrix} x_{\text {fos}}&x_{\text {gas}}&x_{\text {nuc}}&x_{\text {hfc}}&x_{\text {wnd}}&x_{\text {spv}}&x_{\text {sth}}&x_{\text {hyd}}&x_{\text {geo}} \end{bmatrix}^\top \, \end{aligned}$$

#### Parameters

Input data is specific to the region of interest, over a predetermined timespan. These are classified as general and per source parameters, as shown in Table 2. Most of these terms are standard inputs for energy system optimization models, representing the economic and technical characteristics of the available technologies. A key feature of this formulation, however, is the inclusion of the binary parameter vector $\textbf{b}$. This vector acts as a set of “on/off” switches for each energy source, enabling the model to rigorously explore and find the optimal mix for any possible combination of technologies. This transforms the formulation into a powerful tool for scenario analysis.

For instance, to find the optimal mix for a “renewables-only” future that excludes all fossil fuels and nuclear power, the vector $\textbf{b}$ would be set as follows (in the order of $\mathcal {S}$):

3 $$\begin{aligned} \textbf{b} = \begin{bmatrix} \underbrace{0}_{\text {fos}}&\underbrace{0}_{\text {gas}}&\underbrace{0}_{\text {nuc}}&\underbrace{1}_{\text {bes}}&\underbrace{1}_{\text {wnd}}&\underbrace{1}_{\text {spv}}&\underbrace{1}_{\text {sth}}&\underbrace{1}_{\text {hyd}}&\underbrace{1}_{\text {geo}} \end{bmatrix}^\top \, \end{aligned}$$

Conversely, to evaluate a scenario that includes nuclear power but excludes fossil fuels and natural gas, the vector would be set to $$\textbf{b}=\begin{bmatrix} 0&0&1&1&1&1&1&1&1\end{bmatrix}^\top$$. By systematically varying $\textbf{b}$, one can compare the optimal solutions under different policy or technology availability assumptions.

**Table 2:** Input parameters specific to the region of interest for solving the NLP.

Parameter	Description	Units	Type
*General*			
*Y*	Years of operability	years	Constant
$$D^{\text {peak}}$$	Peak power demand	kW	Constant
$$\text {LCOE}^{\text {tar}}$$	Target LCOE	$/kWh	Constant
*p*	Power reserve percentage	–	Constant
*Per energy source*			
$$P^{\text {pot}}_s$$	Potential power capacity	kW	$$N_S\times 1$$ vector
$$C^{\text {cap,ref}}_s$$	Capex of reference plant	$/kW	$$N_S\times 1$$ vector
$$C^{\text {opf,ref}}_s$$	Opex (fixed) of reference plant	$/kW-yr	$$N_S\times 1$$ vector
$$C^{\text {opv,ref}}_s$$	Opex (variable) of reference plant	$/kWh	$$N_S\times 1$$ vector
$$P^{\text {ref}}_s$$	Power reference plant	kW	$$N_S\times 1$$ vector
$$b_s$$	Inclusion in the mix	Bool	$$N_S\times 1$$ vector
$$\kappa _s$$	Capacity factor	–	$$N_S\times 1$$ vector
$$r_s$$	Discount rate	–	$$N_S\times 1$$ vector
$$\alpha _s$$	Cost scaling factor for $$C^{\text {cap}}$$	–	$$N_S\times 1$$ vector
$$\beta _s$$	Cost scaling factor for $$C^{\text {op,fix}}$$	–	$$N_S\times 1$$ vector
$$g_s$$	Fuel price	$/MBtu	$$N_S\times 1$$ vector
$$h_s$$	Heat rate	MBtu/kWh	$$N_S\times 1$$ vector

#### Objective function

The primary objective is to minimize the total Net Present Cost (NPC) of the energy system. This cost function, denoted $C(\textbf{x})$, aggregates initial capital expenditures with the present value of all fixed operational costs over the system’s lifespan, *Y*. This method provides a consistent financial basis for comparing technologies with different cost structures, such as capital-intensive renewables versus fuel-dependent thermal plants. The objective function is formulated as:

4 $$\begin{aligned} \min _{\textbf{x}} C(\textbf{x}) = \sum _{s \in \mathcal {S}} x_s b_s \left( C_s^{\text {cap}} + C_s^{\text {opf}} \cdot A_s \right) \end{aligned}$$

where $A_s$ is the present value annuity factor, which converts a series of future annual payments into a single lump sum in today’s money. It is defined as:

5 $$\begin{aligned} A = \frac{1 - (1+r_s)^{-Y}}{r_s} \end{aligned}$$

In the objective function, the total cost is summed over all sources $$s \in \mathcal {S}$$. For each source, its contribution is determined by its installed capacity $$x_s$$, its specific costs, and the binary parameter $$b_s$$ that dictates its inclusion in the energy mix. The terms $$C_s^{\text {cap}}$$ and $$C_s^{\text {opf}}$$ represent the specific capital ($/kW) and fixed operational ($/kW-yr) costs, respectively. To model economies of scale, these costs are not fixed but are functions of the installed capacity $$x_s$$. This dependency introduces non-linearity into the problem. The explicit mathematical expressions for these expenditures will be defined in the following section on constraints.

#### Constraints

The first constraint (6) is related to the power balance of the system, often called a planning reserve margin or capacity adequacy constraint, which ensures that the total installed capacity is sufficient to meet the peak demand.

6 $$\begin{aligned} p D^{\text {peak}} \le \sum _{s \in \mathcal {S}} x_s b_s \, \end{aligned}$$

The power-reserve percentage *p*, ensures that total dispatchable generation exceeds instantaneous demand by a fixed margin, thereby preserving reliability under renewable variability. The next constraint (7) ensures that the target Levelized Cost of Energy (LCOE) is met. For simplicity, let us define $P_s=x_sb_s\kappa _s$ as the average power served (kW) by source *s*. The total, weighted LCOE is

7 $$\begin{aligned} \sum _{s \in \mathcal {S}} w_s \cdot \text {LCOE}_s \le \text {LCOE}^{\text {tar}}\, \end{aligned}$$

where $w_s$ is the energy share of each source in the final mix. That is

8 $$\begin{aligned} w_s = \frac{P_s}{\sum _{j \in \mathcal {S}} P_j}, \qquad \forall s \in \mathcal {S} \, \end{aligned}$$

The LCOE ($/kWh) for each source is computed as

9 $$\begin{aligned} \text {LCOE}_s=\frac{f_sC^{\text {cap}}_s+C^{\text {opf}}_s}{8760\kappa _s}+C^{\text {opv}}_s+C^{\text {fuel}}_s \,, \qquad \forall s \in {\textbf {S}}\, \end{aligned}$$

where $f_s$ is the fixed charge rate, which annualizes the capital costs of an energy project based on a discount rate *r* and number of periods (years) *Y*. It is defined as

10 $$\begin{aligned} f_s = \frac{r_s(1+r_s)^Y}{\left[ (1+r_s)^{Y}-1\right] }\,, \qquad \forall s \in {\textbf {S}}\, \end{aligned}$$

Note that $$f_s$$ includes the project’s lifetime (*Y*) in its calculation. Thus, multiplying $$f_sC^{\text {cap}}_s$$ effectively spreads the capital expenditure, i.e., the lump-sum overnight cost, out over the project’s lifetime, converting it to an annual expense. The resulting units are $$\$\text {/kW-yr}$$. The capital expenditures $$C^{\text {cap}}_s$$ ($/kW) are the costs required to build or buy a power plant. These costs are scaled to the desired power output $$x_s$$ based on a reference plant with a known power output $$P_s^{\text {ref}}$$ and capital cost $$C_s^{\text {cap, ref}}$$. A scaling factor $$\alpha _s$$ is applied to account for economies of scale.

11 $$\begin{aligned} C^{\text {cap}}_s = C^{\text {cap,ref}}_s \left( \frac{x_sb_s}{P_s^{\text {ref}}} \right) ^{\alpha _s}, \qquad \forall s \in {\textbf {S}}\, \end{aligned}$$

Fixed O&M expenditures $$C^{\text {opf}}_s$$ ($/kW-yr) may be scaled similarly as capital expenditure; however, the scaling factor $$\beta _s$$ is often higher and difficult to estimate for varied energy sources.

12 $$\begin{aligned} C^{\text {opf}}_s = C^{\text {opf,ref}}_s \left( \frac{x_sb_s}{P_s^{\text {ref}}} \right) ^{\beta _s}\,, \qquad \forall s \in {\textbf {S}}\, \end{aligned}$$

Variable O&M expenditures $$C^{\text {opv}}_s$$ ($/kWh) are assumed to scale directly with energy generation. Thus, these rates are not dependent on the power output of the plant.

13 $$\begin{aligned} C^{\text {opv}}_s=C^{\text {opv,ref}}_s\,, \qquad \forall s \in {\textbf {S}}\, \end{aligned}$$

Finally, fuel costs $$C^{\text {fuel}}$$ ($/kWh) depend on the fuel price $$g_s$$ (in $$\$\text {/MBtu}$$) and the heat rate $$h_s$$ (in $$\text {MBtu/kWh}$$) of the plant. For simplicity, these two are considered constants.

14 $$\begin{aligned} C_s^{\text {fuel}}= g_s h_s\,, \qquad \forall s \in {\textbf {S}}\, \end{aligned}$$

#### Bounds

The plant sizes of each source must not exceed the technical capacity specified from the input data.

15 $$\begin{aligned} 0 \le x_s \le b_sP^{\text {pot}}_s \quad \forall s \in \textbf{S}\, \end{aligned}$$

#### Implementation of the model

The formulation presented here constitutes a Non-Linear Programming (NLP) problem. The non-linearity arises primarily from two sources: (1) The economies of scale. The capital and fixed operational costs are modeled as power functions of the decision variables $x_s$ with scaling exponents $\alpha _s$ and $\beta _s$. This captures the real-world phenomenon where the per-unit cost of a larger plant is typically lower than that of a smaller one. (2) The weighted LCOE constraint. The target LCOE constraint is a weighted average where the weights, $w_s$, are themselves functions of the decision variables $x_s$. This introduces a complex rational function into the constraints. Consequently, the adoption of an NLP framework is driven strictly by the need to capture these specific non-linear economic behaviors and does not imply a general superiority over established linear or mixed-integer formulations widely used in the literature. A key simplification in this analysis, adopted due to the difficulty in obtaining precise data for every technology, is the assumption that the cost scaling factors are equal, i.e., $\alpha =\beta =0.6$ for all sources $s\in \textbf{S}$. This value is based on the widely recognized six-tenths rule^29^, an engineering rule of thumb used for estimating the capital cost of industrial plants and equipment. It serves as a reasonable approximation for economies of scale in the absence of more detailed, technology-specific cost-scaling data.

Furthermore, this initial formulation adopts a high-level approach by using an annualized capacity factor, $\kappa$, for each technology. This parameter represents the *average annual* energy output relative to its maximum possible output, effectively neglecting time-dependent fluctuations in energy demand and generation. While a time-series analysis is required for operational dispatch and unit commitment, the proposed method focuses on optimal capacity sizing by capturing non-linear economic efficiencies. This significantly reduces model complexity and computational burden, making it well-suited for high-level scenario planning and initial capacity-expansion analysis. Future iterations of this work could utilize these sized capacities as inputs for dynamic operational models that consider time-dependent load profiles and State of Charge (SoC) dynamics.

Acquiring and processing the remaining input parameters for the model is a critical and often challenging task. The required data is typically scattered across various sources, including governmental reports, academic literature, and industry datasets, each with its own scope, methodology, and time frame. Reconciling these disparate sources to create a consistent and reliable set of input parameters is essential for the model’s validity. Techno-economic parameters relevant for the model were obtained from reliable online sources such as the International Energy Agency and the World Bank’s Global Dataset on Levelized Cost of Energy Generation^30–32^. The exact dataset used to generate these results will be made available upon request.

Figure 2 illustrates the computational workflow for evaluating candidate hybrid energy configurations. Feasible technology combinations are optimized using nonlinear programming. Buckingham–$\pi$ dimensional analysis then computes the 3R energy security metrics (reliability, resilience, and renewability), which determine whether configurations are accepted or re-optimized. The resulting dataset is used to train a Gaussian Process Regression (GPR) surrogate model, and SHAP analysis quantifies the contribution of individual technologies to system performance.

Algorithm 1 summarizes the computational procedure used to generate optimal energy-mix configurations and evaluate their techno-economic and energy security performance metrics.

**Algorithm 1:**
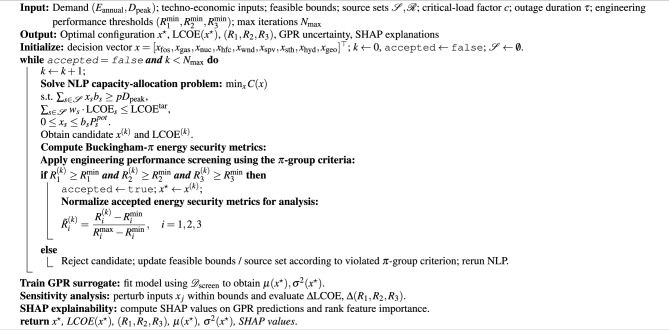
Optimization algorithm for the proposed N-RHES framework

### Aggregation of energy security features and variable selection

Nuclear–Renewable Hybrid Energy Systems (N-RHES) involve numerous design, operational, and environmental parameters that collectively influence reliability, resilience, and sustainability. To enable a tractable dimensional-scaling formulation, key energy security characteristics are systematically aggregated into a compact set of physically interpretable system-level descriptors that preserve the dominant system behavior.

Fifteen critical hybrid-energy system features including generation adequacy, renewable intermittency, ramp flexibility, storage dynamics, peak-demand stress, demand growth uncertainty, outage duration severity, restoration delays, critical-load priority, renewable capacity scale, technology mix, fuel logistics exposure, reserve margin requirements, recovery constraints, and emergency operational flexibility are consolidated into six retained variables. The aggregation follows engineering-consistent system-reduction principles: additive composition preserves conservation relationships for power and energy quantities; peak-envelope operators capture demand and disturbance extremes that define security thresholds; and priority-weighted scaling represents critical infrastructure protection requirements^33,34^.

The resulting reduced variable set consists of system supply capacity $P_s$, peak electricity demand $D_{\textrm{peak}}$, available battery storage energy $E_{\textrm{bes}}$, disturbance duration $\tau$, renewable generation contribution $P_R$, and the critical-load fraction *c*. These six variables form a dimensionally sufficient and physically meaningful basis for constructing the proposed Buckingham-$\pi$ energy security metrics. Considering that the retained variables represent system-level physical quantities rather than location-specific parameters, the resulting dimensionless $\pi$-group formulation is inherently transferable to other islanded power systems and hurricane-prone Caribbean grids with similar operational characteristics.

Table 3 summarizes how the fifteen energy security drivers documented in the literature are mapped to the retained scaling variables for Puerto Rico’s hurricane-prone island grid. The inclusion of supporting references substantiates both the aggregation principles and the selection of variables, ensuring that the proposed dimensional-scaling framework is grounded in established reliability, resilience, and hybrid energy-system research.

**Table 3:** Aggregation of N-RHES energy security drivers into the retained Buckingham-$\pi$ scaling variables for Puerto Rico and hurricane-prone islanded systems.

Hybrid-energy system features	Puerto Rico / hurricane-zone relevance	$P_s$	$D_{\textrm{peak}}$	$E_{\textrm{bes}}$	$\tau$	$P_{\textrm{R}}$	*c*	Literature basis
Generation adequacy	Islanded grid with low tolerance for supply shortfall	$\checkmark$						Power-system reliability & adequacy studies^7,9,10,25,28^
Renewable intermittency	Weather-driven variability after storms	$\checkmark$				$\checkmark$		Renewable integration & variability^6,21,24,35^
Ramp flexibility	Fast demand swings require flexible response	$\checkmark$		$\checkmark$				Grid flexibility & ramping constraint studies^10,11,28^
Storage dynamics	Needed for ride-through and backup autonomy			$\checkmark$				Energy storage & microgrid resilience research^7,26,27^
Peak-demand stress	Extreme-event load surges strain grid stability		$\checkmark$					Peak-load planning & adequacy standards^7,9,32^
Demand growth uncertainty	Long-term planning under volatile consumption trends		$\checkmark$					Load forecasting & planning uncertainty studies^10,12^
Outage duration severity	Storm damage causes prolonged blackouts				$\checkmark$			Disaster energy resilience & outage modeling^16–18^
Restoration delays	Slow grid recovery in disaster aftermath				$\checkmark$		$\checkmark$	Post-hurricane grid recovery & infrastructure restoration reports^23,24^
Critical-load priority	Hospitals, water, telecom must remain powered						$\checkmark$	Critical infrastructure protection & resilience frameworks^7,9,24^
Renewable capacity scale	Expansion planning for renewable deployment					$\checkmark$		Renewable capacity planning & hybrid energy studies^6,21,35^
Technology mix	Nuclear–renewable balance affects security	$\checkmark$				$\checkmark$		Hybrid energy systems & diversification literature^11,12,25^
Fuel logistics exposure	Import and port disruptions threaten supply	$\checkmark$						Island energy security & fuel dependency reports^15,16,21^
Reserve margin needs	Islanded grids require added operating buffers	$\checkmark$	$\checkmark$					Reliability standards & reserve margin criteria^7,9,28^
Recovery constraints	Post-disaster endurance requirements			$\checkmark$	$\checkmark$		$\checkmark$	Infrastructure resilience & emergency energy planning^16–18,24^
Emergency operational flexibility	Fast-response capability during cascading failures	$\checkmark$		$\checkmark$	$\checkmark$		$\checkmark$	Grid resilience & emergency operations literature^10,28^

### Scaling of energy security metrics using the Buckingham–$\pi$ theorem

The scaling of energy security metrics provides a systematic framework for evaluating hybrid energy systems in a manner that is independent of system size or configuration. In this study, the three energy security metrics (reliability, resilience, and renewability) are derived using the Buckingham $\pi$-theorem, a classical dimensional analysis method widely used to obtain scale-independent performance indicators^36^.

Nuclear-Renewable Hybrid Energy Systems contain numerous technical, operational, and economic variables. However, their macroscopic behavior can be represented using a compact set of system-level scaling variables that capture the dominant physical constraints governing supply adequacy, storage endurance, renewable penetration, and disturbance response. Based on the aggregation described in the previous section, the hybrid system is represented using six physically meaningful variables that span the fundamental quantities of power, energy, and time. These quantities form a dimensionally complete basis from which the dimensionless performance metrics are derived.

Applying the Buckingham $\pi$-theorem allows the dimensional variables to be transformed into scale-invariant groups that preserve the underlying physical relationships while reducing model complexity. This dimensional reduction enables meaningful comparison across diverse hybrid energy architectures and different system scales.

The six relevant variables are defined as:

16 $$\begin{aligned} \{P_s,\, D_{\text {peak}},\, E_{\text {bes}},\, \tau ,\, P_R,\, c\} \;\rightarrow \; \{\Pi _1,\, \Pi _2,\, \Pi _3\} \end{aligned}$$

where $P_s$ denotes the power output of source *s*, $D_{\textrm{peak}}$ represents the peak electricity demand, $E_{\textrm{bes}}$ is the available battery energy storage capacity, and $\tau$ denotes the reference outage duration. The renewable power contribution is denoted as:

17 $$\begin{aligned} P_R=\sum _{s\in \mathscr {R}}P_s \end{aligned}$$

while the total installed generation capacity is:

18 $$\begin{aligned} P_{\textrm{tot}}=\sum _{s\in \mathscr {S}} P_s \end{aligned}$$

where $\mathscr {S}$ denotes the set of all generation sources and $\mathscr {R}$ represents the subset of renewable technologies. The parameter *c* represents the dimensionless critical-load fraction.

The dimensional form of these variables is:

19 $$\begin{aligned} \left\{ \begin{aligned} [P_s]&= [M^1 L^2 T^{-3}] \\ [D_{\text {peak}}]&= [M^1 L^2 T^{-3}] \\ [E_{\text {bes}}]&= [M^1 L^2 T^{-2}] \\ [\tau ]&= [M^0 L^0 T^{1}] \\ [P_R]&= [M^1 L^2 T^{-3}] \\ [c]&= [M^0 L^0 T^0] \end{aligned} \right. \end{aligned}$$

Since the six variables span the three fundamental dimensions (*M*, *L*, *T*), the Buckingham $\pi$-theorem yields three independent dimensionless groups.

The power variables $\{P_s, P_R, D_{\text {peak}}\}$ share the same physical dimension and therefore are not dimensionally independent. A single representative variable, $D_{\text {peak}}$, is retained to represent the power scale, while $\tau$ represents the time scale. The repeating variables are therefore selected as:

20 $$\begin{aligned} \{D_{\text {peak}},\;\tau \}, \end{aligned}$$

which together span the independent dimensions required to normalize the remaining variables and construct the dimensionless $\pi$-groups.

Reliability metric $\Pi$-group

The first $\pi$-group is constructed from the system power supply variable $P_s$:

21 $$\begin{aligned} \Pi _1=P_s \cdot D_{\text {peak}}^{a}\cdot \tau ^{b} \end{aligned}$$

Substituting dimensions,

22 $$\begin{aligned} [\Pi _1]=(ML^2T^{-3})(ML^2T^{-3})^{a}(T)^{b} = M^{1+a}L^{2+2a}T^{-3-3a+b} \end{aligned}$$

For dimensional closure,

23 $$\begin{aligned} M^{1+a}L^{2+2a}T^{-3-3a+b}=M^0L^0T^0 \end{aligned}$$

which yields

24 $$\begin{aligned} \left\{ \begin{aligned} 1+a&=0 \\ 2+2a&=0 \\ -3-3a+b&=0 \end{aligned} \right. \Rightarrow a=-1,\quad b=0 \end{aligned}$$

Thus,

25 $$\begin{aligned} \Pi _1=\frac{P_s}{D_{\text {peak}}} \end{aligned}$$

At the system-level the reliability metric becomes

26 $$\begin{aligned} R_1=\frac{\sum _{s\in S}P_s}{D_{\text {peak}}} \end{aligned}$$

which represents the ratio between total available generation capacity and peak system demand.

Resilience metric $\Pi$-group

The resilience $\pi$-group is constructed using the storage energy variable $E_{\text {bes}}$:

27 $$\begin{aligned} \Pi _2 = E_{\text {bes}} \cdot D_{\text {peak}}^a \cdot \tau ^b \cdot c^e \end{aligned}$$

Substituting dimensions:

28 $$\begin{aligned} [ML^2T^{-2}] [ML^2T^{-3}]^{a} [T^b] [1]^e = [M^0 L^0 T^0] \end{aligned}$$

which gives

29 $$\begin{aligned} \left\{ \begin{aligned} 1 + a&= 0 \\ 2 + 2a&= 0 \\ -2 - 3a + b&= 0 \end{aligned} \right. \Rightarrow a=-1,\; b=-1 \end{aligned}$$

Since the critical-load fraction *c* is dimensionless, it does not influence dimensional closure but is retained to represent the fraction of peak demand that must be sustained during disruptions. Taking $e=-1$, the resilience group becomes:

30 $$\begin{aligned} \Pi _2=\frac{E_{\text {bes}}}{cD_{\text {peak}}\tau } \end{aligned}$$

and the system-level resilience metric is:

31 $$\begin{aligned} R_2=\frac{E_{\text {bes}}}{cD_{\text {peak}}\tau } \end{aligned}$$

which measures the capability of storage resources to sustain critical demand during outages.

Renewability metric $\Pi$-group

The renewable $\pi$-group is obtained from the renewable power contribution $P_R$ as:

32 $$\begin{aligned} \Pi _R=P_R \cdot \ D_{\text {peak}}^{a}\cdot \ \tau ^{b} \end{aligned}$$

Substituting dimensions:

33 $$\begin{aligned} [\Pi _R]=(ML^2T^{-3})(ML^2T^{-3})^{a}(T)^{b} = M^{1+a}L^{2+2a}T^{-3-3a+b} \end{aligned}$$

For dimensional closure,

34 $$\begin{aligned} M^{1+a}L^{2+2a}T^{-3-3a+b}=M^0L^0T^0, \end{aligned}$$

yielding:

35 $$\begin{aligned} \left\{ \begin{aligned} 1+a&=0,\\ 2+2a&=0,\\ -3-3a+b&=0. \end{aligned} \right. \Rightarrow a=-1,\; b=0 \end{aligned}$$

From system-level implementation:

36 $$\begin{aligned} \Pi _R=\frac{P_R}{D_{\text {peak}}} \Rightarrow \frac{\sum _{s\in \mathscr {R}}P_s}{D_{\text {peak}}} \end{aligned}$$

Thus, the system-level renewability $\Pi$-group metric is obtained as a ratio between two - $\Pi$-groups as:

37 $$\begin{aligned} R_3=\Pi _3 =\frac{\Pi _R}{\Pi _1} =\frac{\left( \sum _{s\in \mathscr {R}}P_s\right) /D_{\text {peak}}}{\left( \sum _{s\in \mathscr {S}}P_s\right) /D_{\text {peak}}} =\frac{\sum _{s\in \mathscr {R}}P_s}{\sum _{s\in \mathscr {S}}P_s} \end{aligned}$$

which represents the fraction of total installed capacity provided by renewable technologies.

The critical demand ratio *c* is defined as:

38 $$\begin{aligned} c = \frac{D_{\text {critical}}}{D_{\text {total}}} \end{aligned}$$

where $D_{\text {critical}}$ represents the essential load that must remain supplied during grid disturbances. This parameter appears explicitly in the resilience metric to quantify the system’s ability to sustain critical infrastructure during outages.

Within the optimization framework developed in this work, the 3R metrics are implemented as complementary optimization performance indicators used to evaluate the robustness of candidate hybrid configurations. This approach allows the primary techno-economic optimization to remain unbiased with respect to capacity scaling and storage sizing, while the dimensionless metrics reveal reliability, resilience, and sustainability trends across the feasible design space. A summary of the resulting dimensionless $\pi$-groups, the complex system features they scale, and their decision relevance is provided in Table 4.

**Table 4:** Buckingham $\pi$-based dimensionless 3R energy security metrics and their decision relevance.

3R metric	$\Pi$-Groups	Complex system features scaled	Decision relevance
*Reliability (R1)*			
Ratio of actual power served to demand	$\displaystyle \Pi _1=\frac{\sum _{s \in \mathscr {S}} P_s}{D_{\textrm{peak}}}$	Multi-source generation mix, aggregate supply adequacy, and peak-demand. $\Pi _1 = f(P_s, D_{\text {peak}})$	Indicates whether total system capacity is sufficient to meet demand
*Resilience (R2)*			
Ratio of available storage energy to critical-load energy demand	$\displaystyle \Pi _2=\frac{E_{\textrm{bes}}}{c\,D_{\textrm{peak}}\,\tau }$	Storage sizing, outage duration, and critical-load survival requirements. $\Pi _2 = f(E_{\text {bes}}, c, \tau , D_{\text {peak}})$	Indicates ability to sustain essential loads during disruptions
*Renewability (R3)*			
Ratio of renewable contributions to total power served	$\displaystyle \Pi _3=\frac{\sum _{s \in \mathscr {R}} P_s}{\sum _{s \in \mathscr {S}} P_s}$	Technology composition and renewable penetration within total installed capacity. $\Pi _3 = f(P_s)$	Indicates level of renewable integration and sustainability.

To evaluate the hybrid-system performance across the candidate configurations, the resulting Buckingham–$\pi$ groups are summarized in Table 4. These dimensionless 3R metrics (reliability, resilience, and renewability) normalize key system variables such as generation capacity, storage energy, outage duration, and renewable penetration, enabling scale-independent comparison of hybrid architectures. In this work, the 3R metrics are incorporated as minimum feasibility thresholds during the optimization process and as system-level performance indicators used to compare the energy security characteristics of the resulting feasible configurations.

### Energy security $\pi$-group metrics evaluation

The dimensionless 3R energy security $\pi$-group metrics complement the nonlinear programming optimization framework by providing engineering screening criteria for selecting acceptable solutions within the feasible design space. During the optimization workflow, the decision variables of each candidate solution are mapped to the corresponding $\pi$-group expressions to evaluate the reliability, resilience, and renewability indicators used to determine whether the energy mix satisfies the required security thresholds.

Let the NLP decision vector collect the installed capacities of all candidate technologies:

39 $$\begin{aligned} \textbf{x} = \begin{bmatrix} x_{\textrm{fos}}&x_{\textrm{gas}}&x_{\textrm{nuc}}&x_{\textrm{bes}}&x_{\textrm{wnd}}&x_{\textrm{spv}}&x_{\textrm{sth}}&x_{\textrm{hyd}}&x_{\textrm{geo}} \end{bmatrix}^{\!\top } \end{aligned}$$

where $x_{\textrm{bes}}$ denotes the generalized bulk energy storage variable representing the hydrogen fuel-cell storage subsystem defined in the Methods section.

The diagonal availability (capacity-factor) matrix is defined as:

40 $$\begin{aligned} \textbf{K} = \textrm{diag} (\kappa _{\textrm{fos}}, \kappa _{\textrm{gas}}, \kappa _{\textrm{nuc}}, \kappa _{\textrm{bes}}, \kappa _{\textrm{wnd}}, \kappa _{\textrm{spv}}, \kappa _{\textrm{sth}}, \kappa _{\textrm{hyd}}, \kappa _{\textrm{geo}}) \end{aligned}$$

The average available power from each technology is given by the relation:

41 $$\begin{aligned} \textbf{p}(\textbf{x}) = \textbf{K}\textbf{x} \end{aligned}$$

Let $\textbf{e}_{\textrm{bes}}$ denote the canonical selector vector associated with the storage component and $\textbf{e}_{\textrm{ren}}$ the selector vector for renewable technologies: $\mathcal {R}=\{\textrm{wnd},\textrm{spv},\textrm{sth},\textrm{hyd},\textrm{geo}\}$.

Reliability ($R_1$) The reliability $\pi$-metric measures the ratio of total available generation capacity to peak system demand as:

42 $$\begin{aligned} R_1(\textbf{x}) = \frac{\textbf{1}^{\top }\textbf{p}(\textbf{x})}{D_{\textrm{peak}}} \end{aligned}$$

Resilience ($R_2$) The resilience $\pi$-metric quantifies the capability of dispatchable storage to sustain critical loads during disturbances. The available storage contribution is computed as:

43 $$\begin{aligned} E_{\textrm{bes}}(\textbf{x}) = \textbf{e}_{\textrm{bes}}^{\top }\textbf{p}(\textbf{x}) = \textbf{e}_{\textrm{bes}}^{\top }\textbf{K}\textbf{x} \end{aligned}$$

The critical load is defined using the peak system demand $D_{\textrm{peak}}$ and the critical-load fraction *c*.

44 $$\begin{aligned} P_{\textrm{crit}} = c\,D_{\textrm{peak}} \end{aligned}$$

The resilience metric is also vectorized as:

45 $$\begin{aligned} R_2(\textbf{x}) = \frac{E_{\textrm{bes}}(\textbf{x})}{c\,D_{\textrm{peak}}\tau } \end{aligned}$$

where $\tau$ denotes the disturbance duration.

Renewability ($R_3$) The renewability $\pi$-metric represents the fraction of renewable generation within the total power mix as:

46 $$\begin{aligned} R_3(\textbf{x}) = \frac{\textbf{e}_{\textrm{ren}}^{\top }\textbf{p}(\textbf{x})}{\textbf{1}^{\top }\textbf{p}(\textbf{x})} \end{aligned}$$

The NLP optimization is solved over the feasible region:

47 $$\begin{aligned} \mathcal {F} = \left\{ \textbf{x} \in \mathbb {R}^{N_s} : \underbrace{\textbf{1}^{\top }\textbf{x} \ge pD_{\textrm{peak}}}_{\text {capacity adequacy}} \hspace{0.2cm}, \; \underbrace{\textbf{w}^{\top }(\textbf{x})\,\boldsymbol{\ell }(\textbf{x}) \le \textrm{LCOE}_{\textrm{tar}}}_{\text {LCOE constraint}} \hspace{0.2cm}, \; \underbrace{\textbf{0} \le \textbf{x} \le \textbf{b} \odot \textbf{P}^{\textrm{pot}}}_{\text {bounds}} \right\} \end{aligned}$$

with optimal solution:

48 $$\begin{aligned} \textbf{x}^{\star } = \arg \min _{\textbf{x}\in \mathcal {F}} C(\textbf{x}) \end{aligned}$$

After obtaining $\textbf{x}^{\star }$, the reported energy security metrics are evaluated by direct substitution as:

49 $$\begin{aligned} R_i^{\star } = R_i(\textbf{x}^{\star }), \qquad i=1,2,3 \end{aligned}$$

The computed $\pi$-group metrics are then used as engineering screening criteria consistent with Algorithm 1. If the optimized solution satisfies the minimum security thresholds $R_1 \ge R_1^{\min }$, $R_2 \ge R_2^{\min }$, and $R_3 \ge R_3^{\min }$, the candidate energy mix is accepted for further techno-economic analysis. Otherwise, the violated $\pi$-group criterion is used to adjust the source bounds or technology availability limits, and the NLP optimization is repeated until an acceptable solution is obtained as detailed in Algorithm 1.

### Water consumption

The analysis incorporated water consumption as a vital sustainability indicator, especially for island regions such as Puerto Rico that suffer from periodic droughts and climate-related stress. To quantify the annual water consumption across different energy configurations, the following mathematical relation was applied:

50 $$\begin{aligned} \text {Water}_{\text {consumed}} \; [\text {gal/year}] = \sum _{h=1}^{8760} \left( \frac{E_h \; [\text {MWh}]}{\text {Water Intensity} \; [\text {MWh/gal}]} \right) = \sum _{h=1}^{8760} \left( \frac{E_{\text {annual}}/8760 \; [\text {MWh}]}{\text {Water Intensity} \; [\text {MWh/gal}]} \right) \end{aligned}$$

where $\text {Water}_{\text {consumed}}$ refers to total annual water use (in gallons) for a particular energy configuration, $E_h$ denotes hourly electricity generation (in MWh), and is assumed as $E_{\text {annual}}/8760$ based on Puerto Rico’s projected annual demand. Lastly, the term ‘Water Intensity’ refers to the specific energy consumption per gallon of water which varies by technology: geothermal, nuclear, or hydrogen-based systems have considerably higher water intensity compared to solar PV and wind systems. This approach makes it possible to calculate the specific configuration’s water consumption aligned precisely with energy production estimates.

Hourly energy production for estimating water consumption was derived from the annual generation term $E_{\text {annual}}/8760$, which assumes constant output throughout the year. This steady-state simplification facilitates cross-technology comparison without intermittency of renewable resources such as solar and wind, potentially smoothing the variability that links electricity generation to water withdrawal and consumption. Empirical data show that water-use intensity differs by up to three orders of magnitude—from less than 300 L/MWh for wind and solar PV to over 2000 L/MWh for thermoelectric plants with closed-loop cooling, depending on regional climate and cooling design^37,38^. Accordingly, the bias introduced by the relation (50) approach is minor for low-water-use renewables but more pronounced for water-intensive thermal technologies not considered in this study.

### Return on investment analysis

The Return on Investment (ROI) serves as an imperative financial metric in evaluating the economic feasibility of each proposed energy configuration. ROI evaluates the efficiency of an investment by comparing the net returns over the project’s operational life to the initial capital expenditure. For this study, ROI is computed as:

51 $$\begin{aligned} \text {ROI} = \frac{\sum _{t=1}^{Y} \left( R_t - C_t \right) }{C_{\text {initial}}} \end{aligned}$$

where $R_t$ denotes the annual revenue or cost savings in year $t$, $C_t$ represents the annual operational and maintenance (O&M) costs, and $C_{\text {initial}}$ is the upfront capital expenditure. The analysis assumes a project lifetime of $Y$ years. In general, the ROI indicates the economic viability of the energy system, which is particularly important for small island economies such as Puerto Rico that require long-term financial sustainability alongside energy reliability. While LCOE captures unit energy cost, ROI complements it by addressing overall investment efficiency and return potential.

###  Sensitivity and interpretability using Gaussian process regression

To support sensitivity analysis and interpretability of the model, a GPR surrogate model has been implemented along with the primary NLP optimization model. GPR is a non-parametric, probabilistic learning method that models a distribution over functions, allowing it to effectively capture uncertainties and nonlinearities within the optimization space. In this study, GPR was trained on the results of the energy mix optimization simulations to approximate the mapping between techno-economic input variables such as capital cost, capacity factor, and installed capacity and critical performance metrics, including LCOE and the 3R indicators (Reliability, Resilience, and Renewability).

SHAP (SHapley Additive exPlanations)^39^ was applied to interpret the Gaussian Process Regression (GPR) surrogate model through feature attribution, providing transparent insight into variable contributions. The SHAP importance and partial-dependence behavior reveal that nuclear capacity and battery energy storage contribute most strongly to reductions in LCOE, underscoring their critical role in lowering system costs when integrated within hybrid energy configurations.

On the other hand, wind energy demonstrated positive SHAP values at high capacity levels which indicate greater costs or reduced return variability. For metrics of energy security, the analysis showed that for Resilience (R2), battery storage and hydrogen fuel cells dominated, while solar thermal and wind energy most impacted Renewability (R3), as expected. This analysis enabled a deeper understanding of the drivers of system performance and provided transparent insights to guide policy and planning. The integration of GPR and SHAP therefore enhances the decision-support capacity of the optimization framework, allowing robust modeling and explainable AI for energy system design.

Gaussian Process Regression (GPR) model was used to approximate the mapping between techno-economic input variables $\textbf{x} \in \mathbb {R}^{N_s}$ and output response variables *y* (e.g., LCOE, 3R metrics) through function distributions described in Algorithm 2 below, which forms the basis of the GPR surrogate model used for interpretability and sensitivity analysis in this study.

**Algorithm 2:**
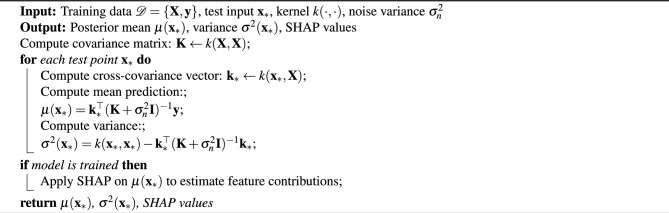
GPR Surrogate Model with SHAP Interpretation

## Results and discussion

We begin by identifying candidate scenarios (referred to as *configurations*) based on the results of the optimization framework and the 3R metrics. According to recent reports, Puerto Rico’s energy demand is estimated at approximately 18 TWh/year, which corresponds to an average power consumption of $\approx 2$ GW. However, optimization problems involving variables at the gigawatt scale often suffer from numerical ill-conditioning, where large magnitude differences between cost coefficients and power variables can lead to solver instability. To ensure robust convergence, we employed a per-unit optimization approach by scaling the demand profile and all power-capacity decision variables by a constant base value such that the representative peak demand is $D_{\text {peak}} \approx 2$ MW. This “unit-scale” formulation preserves the governing techno-economic ratios (consistent with the dimensionless $\pi$-groups) while avoiding numerical artifacts. The resulting optimal capacity shares are then linearly up-scaled to meet the full island demand. Furthermore, the potential capacity bounds ($P^{\text {pot}}_s$) were normalized to ensure a balanced exploration of the decision space, preventing any single technology from dominating the mix solely due to loose upper bounds. This constraint also avoids placing disproportionate weight on a single renewable source. Despite the abundance of these resources in the Caribbean, physical and legal limitations (e.g., land use, regulations) exist, and these tighter bounds serve to account for those realities.

The inputs for the NLP model are presented in Table 5. The computation of the capital recovery factor ($f_s$), used to calculate the LCOE, is performed over a project lifetime of $Y=25$ years, utilizing a power reserve factor of $p=1.1$. As stated previously, we apply the six-tenths rule for the economies of scale constraints, where $\alpha _s=\beta _s\approx 0.6$. Given that our study focuses on renewable grids with base-load support from nuclear and energy storage, the set of active candidate sources is defined as:

52 $$\begin{aligned} \mathcal {S} = \{ \text {nuc, hfc, wnd, spv, sth, hyd, geo} \}\,, \end{aligned}$$

where we have selected hydrogen-based long-duration storage (hfc) as the representative technology for energy storage. Specific sub-technologies across all energy sources are aggregated into their respective broader categories (e.g., conventional hydroelectric, pumped-storage, and run-of-the-river hydro are represented by the hyd category, while onshore and offshore wind are represented by the wnd category). This ensures that the model can be generalized to other geographical regions and islanded grids, where specific types of renewable sources may be more predominant than others. Moreover, despite the proven vast availability of some resources, such as offshore wind in the PR region, grouping these sub-technologies ensures the optimizer cannot exploit individual upper bounds across multiple sub-categories, enforcing a strictly limited overall availability for each overarching resource class to better reflect realistic deployment scenarios.

Furthermore, within this reduced-scale representative profile, the nuclear source effectively functions as a micro-reactor; however, when scaled to the full island demand, this capacity corresponds to the deployment of SMRs. The algorithm explores multiple combinations of these sources by systematically varying the boolean inclusion vector $\textbf{b}$.

**Table 5:** Techno-economic input parameters for each energy source utilized in the NLP optimization.

Param.	Units	fos	gas	nuc	hfc	wnd	spv	sth	hyd	geo
$$P^{\text {pot}}_s$$	kW	1000	1000	1000	10,000	1000	1000	1,000	1000	1000
$$C^{\text {cap,ref}}_s$$	$/kW	4344	1161	7911	706	2285	1688	7000	3630	4892
$$C^{\text {opf,ref}}_s$$	$/kW-yr	0.01	0.02	0.02	15.00	0.03	0.01	0.01	0.01	0.04
$$C^{\text {opv,ref}}_s$$	$/kWh	0.01	0.00	0.00	–	–	–	–	0.00	0.00
$$P^{\text {ref}}_s$$	kW	400	600	900	150	250	200	180	120	150
$$\kappa _s$$	–	0.75	0.75	0.80	0.08	0.30	0.15	0.55	0.40	0.80
$$r_s$$	–	0.07	0.07	0.07	0.07	0.07	0.07	0.07	0.07	0.07
$$g_s$$	$/MBtu	1.58	3.48	0.74	–	–	–	–	–	–
$$h_s$$	MBtu/kWh	0.01	0.01	0.01	–	–	–	–	–	–

The optimal feasible solution, as returned by the SQP/SLSQP solver, is described in detail, including its capacity allocation, operating profiles, and total cost breakdown. We then examine how its LCOE aligns with the 3R metrics; reliability, resilience, and renewability. A comparison table follows, ranking all hybrid configurations considered and benchmarking their optimized LCOEs against state-of-the-art deployments from the literature. This is complemented by a techno-economic evaluation and an assessment of water usage, highlighting the cooling demands associated with each energy pathway. Finally, a targeted sensitivity analysis using a Gaussian Process Regression (GPR) algorithm quantifies how variations in capital cost, capacity factor, and discount rate affect both LCOE and 3R performance. Collectively, these sections provide further insight into how the proposed methodology arrives at configurations which reduce cost, strengthen energy security, and reduce resource consumption.

**Fig. 3:**
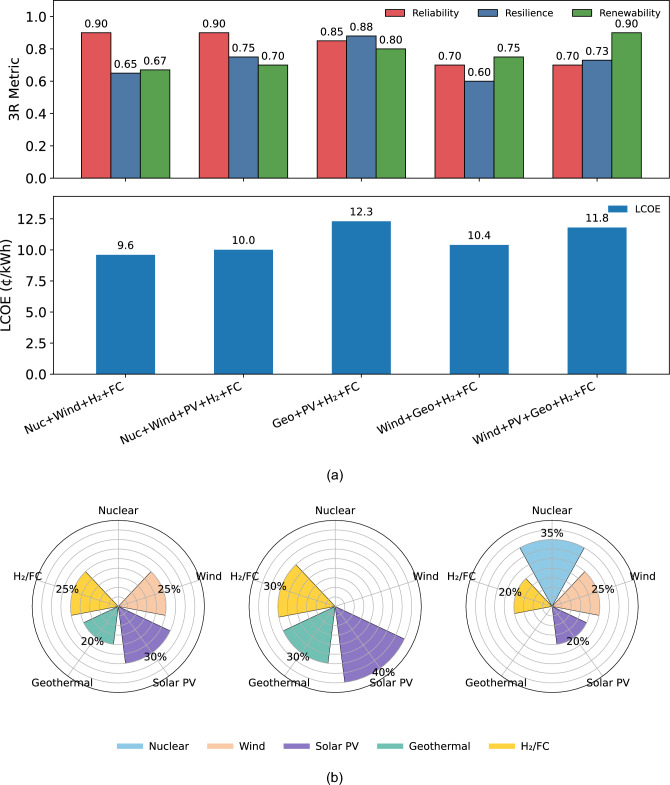
Cost–security and optimized resource mix: (**a**) Bar chart of optimized hybrid configurations with 3R metrics; (**b**) Percentage of each resource in the three best-optimal energy mixes. Left – Wind + Solar PV + Geothermal + H_2_/FC, Middle – Geothermal + Solar PV + H_2_/FC, Right – Nuclear + Wind + Solar PV + H_2_/FC.

**Fig. 4:**
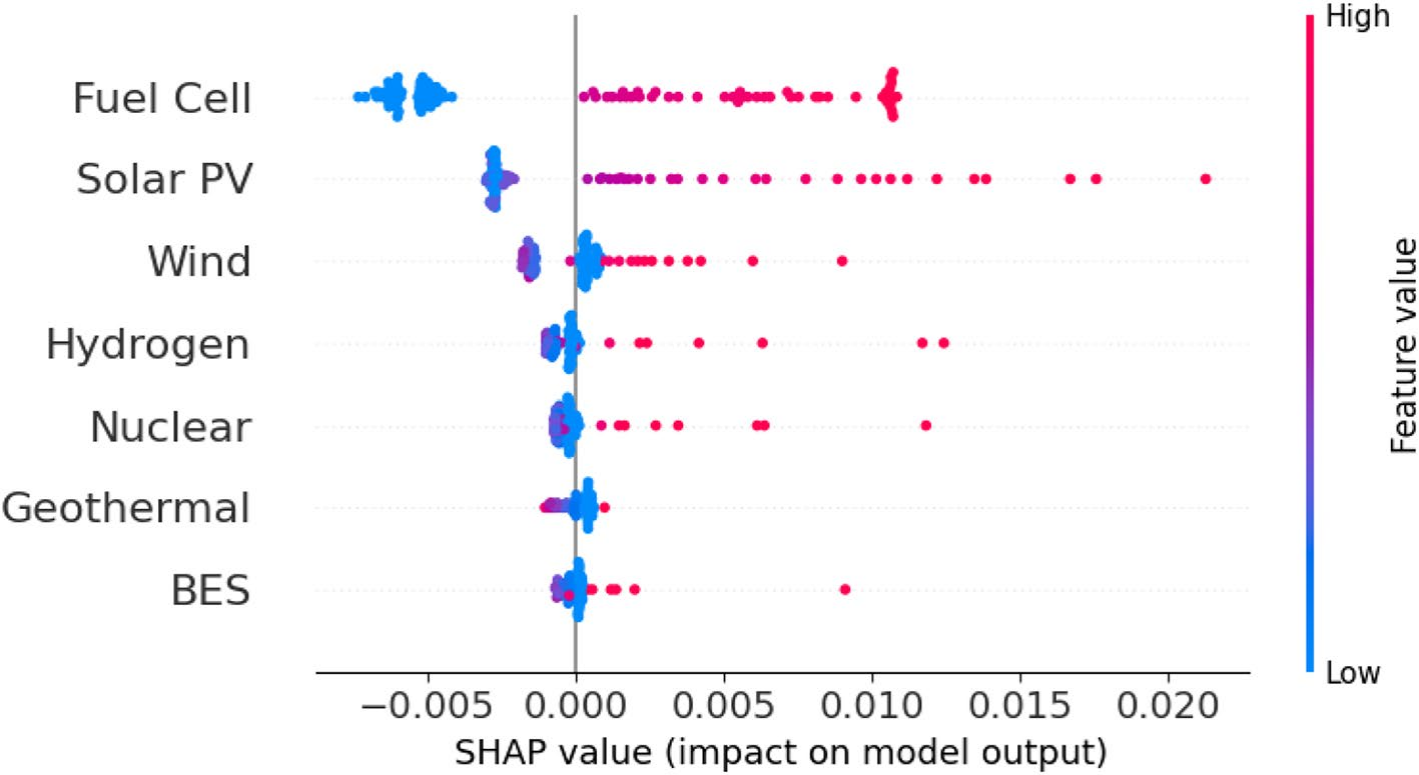
SHAP Impact of energy sources on LCOE.

**Fig. 5:**
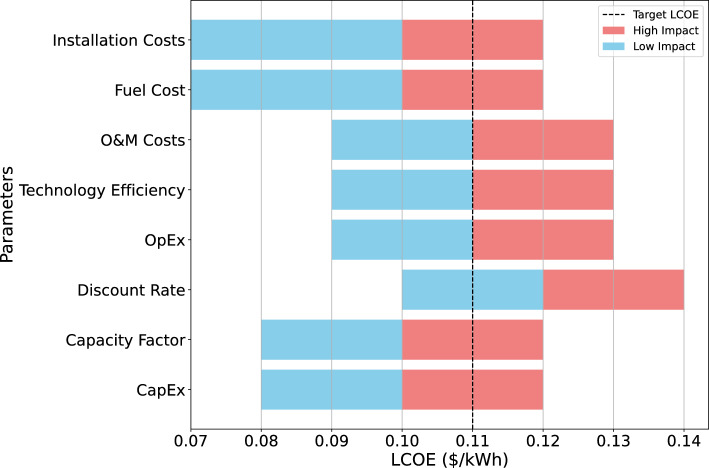
LCOE sensitivity to key techno-economic drivers.

Performance under the optimal hybrid configuration was assessed using multiple quantitative and interpretability-based indicators. The 3R energy security metrics Fig. 3a quantify reliability, resilience, and renewability; LCOE benchmarking Table 6 and ROI–water usage metrics Table 7 capture the economic and environmental performance; while SHAP explainability Fig. 4 and sensitivity analysis Fig. 5 provide insight into the influence of techno-economic and $\pi$-group parameters. Collectively, these evaluations present a holistic view of system performance beyond cost minimization, linking resilience, renewability, and robustness within the optimized configuration.

### LCOE - 3R metrics & resource-mix percentages per configuration

This section presents the results of the optimization framework, illustrating the trade-offs between LCOE and the scaled 3R energy security metrics across five candidate hybrid energy configurations. The optimization identifies five feasible system designs that satisfy the island’s demand and energy security constraints through different combinations of nuclear, wind, solar PV, geothermal, and hydrogen–fuel-cell storage technologies. As shown in Fig. 3a, each configuration exhibits a distinct balance between cost and energy security performance. Collectively, these solutions highlight the strategic choices Puerto Rico faces: a lower-cost pathway incorporating nuclear base-load capacity, a highly resilient renewable-dominant system centered on geothermal and solar resources, or a balanced mid-cost hybrid mix that maintains strong reliability, resilience, and renewability while significantly reducing dependence on imported fossil fuels. Among the five solutions, Configurations 1, 3, and 5 emerge as the most compelling options due to their favorable techno-economic performance and strong 3R security outcomes, representing viable pathways for Puerto Rico’s hybrid energy transition.

Figure 3a presents bar charts of each optimized hybrid energy mix for LCOE (bottom) and energy security (top, represented by the 3R metric scores). In each configuration, it can be observed that:

1. **Configuration 1: Geothermal + Solar PV + H**_2_ **+ FC** tops the table for resilience (0.88) and renewability (0.80) but at the steepest price (${12.3}{\not \! c }/\text {kWh}$); a storm-hardy, carbon-lean choice if budget constraints ease.
2. **Configuration 2: Wind + Geothermal + H**_2_ **+ FC** has an LCOE of ${10.4}{\not \! c }/\text {kWh}$, yet drops resilience to 0.60, revealing the risk of pairing two weather-sensitive sources without a steady nuclear or solar backup.
3. **Configuration 3: Wind + Solar PV + Geothermal + H**_2_ **+ FC** attains the highest renewability (0.90); however, its mid-range cost (${11.8}{\not \! c }/\text {kWh}$) and moderate resilience (0.73) slot it between the budget-friendly nuclear mixes and the premium geothermal–solar option.
4. **Configuration 4: Nuclear + Wind + H**_2_ **+ FC** delivers the lowest LCOE (${9.6}{\not \! c }/\text {kWh}$) with high reliability (0.90). However, its resilience and renewability are relatively low at 0.65 and 0.67, respectively. It is the cheapest hedge against diesel import shocks, though it is only modestly renewable.
5. **Configuration 5: Nuclear + Wind + Solar PV + H**_2_ **+ FC** has a slightly higher LCOE at ${10.0}{\not \! c }/\text {kWh}$ and increased renewability at 0.70 while maintaining high reliability (0.90). This mix forms a balanced firm-plus-variable package for the swing of Puerto Rico’s day–night demand.

Figure 3b shows the optimal energy mix of some select configurations for PR’s projected future energy requirements:

- **Configuration 1:** *Geothermal + Solar PV + H*_2_*/FC* Here the energy mix relies on **40%** solar PV, **30%** geothermal and **30%** H_2_/fuel cells, omitting wind entirely to sidestep hurricane-related turbine outages and coastal siting conflicts. Geothermal’s 24/7 output prevents oversizing hydrogen storage, which helps lower overall cost while preserving a zero-carbon footprint.
- **Configuration 3:** *Wind + Solar PV + Geothermal + H*_2_*/FC* This mix allocates **30%** to solar PV, **25%** to wind power, **20%** to geothermal energy, and **25%** to H_2_/fuel-cell storage, which meets Puerto Rico’s requirement of a fully renewable energy system while enhancing the daily cycle balance by integrating variable renewables with dispatchable geothermal and hydrogen cycling. Since this technology is modular and scalable, this approach may be beneficial for PR’s small island grid when capital budgets are constrained or lagging in transmission upgrades.
- **Configuration 5:** *Nuclear + Wind + Solar PV + H*_2_*/FC* **35%** of the total mix coming from small modular reactors provides a high-capacity-factor backbone when complemented with **25%** wind power, **20%** solar PV and **20%** H_2_/fuel cells storage. The nuclear component steadies both price and supply, allowing smaller hydrogen and renewable capacities to achieve the same reliability target. For an island currently dependent on imported fossil fuels, this nuclear-hybrid alternative not only diversifies fuel sources, but also hedges fuel-price volatility and offers co-benefits such as industrial heat or desalination, all on a footprint smaller than a large solar or wind farm.

Together, Configurations 1, 3, and 5 span a strategic continuum ranging from a cost-stabilizing nuclear hybrid energy mix, through a wind-averse yet carbon-neutral mix, to a wind-geothermal renewable blend—offering Puerto Rico flexible steps toward an affordable, resilient and decarbonized future grid options.

### LCOE benchmarking of candidate hybrid-energy configurations

The five-candidate hybrid-energy configurations that were analyzed, alongside their relative benchmarks in the literature, are presented in the context of levelized cost of energy in Table 6.

**Table 6:** LCOE comparison for optimized hybrid configurations with recent literature.

Configuration	This study LCOE (¢/kWh)	Literature range (¢/kWh)	References
Geothermal + Solar PV + H$\phantom{0}_2$ + FC	12.3	10–18	^40^
Wind + Geothermal + H$\phantom{0}_2$ + FC	10.4	9–17	^41^
Wind + Solar PV + Geothermal + H$\phantom{0}_2$ + FC	11.8	8–16	^42^
Nuclear + Wind + H$\phantom{0}_2$ + FC	9.6	8–15	^43^
Nuclear + Wind + Solar PV + H$\phantom{0}_2$ + FC	10.0	9–16	^44^

These LCOE ranges were derived by aggregating cost data from the individual technologies used in each configuration based on reports from the respective referenced sources. The obtained LCOEs range from 9.6 ¢/kWh for the Nuclear + Wind + H$\phantom{0}_2$ + Fuel Cell (FC) option to 12.3 ¢/kWh for the Geothermal + Solar PV + H$\phantom{0}_2$ + FC configuration. This comparison highlights the competitiveness of each configuration. It also reveals that solar- and geothermal-heavy systems tend to yield LCOE values near the middle to upper end of the range, while nuclear-based hybrids consistently fall closer to the lower bound, reflecting their stronger economic viability.

Configurations that combine a firm low-carbon base-load like nuclear or geothermal with variable renewables plus hydrogen-enabled storage reach LCOE levels that are even below the lower bound of existing literature. This indicates that clean, diversified source alternatives can be more cost-effective. Furthermore, the narrowing spread between our results and the literature indicates that further declines in the cost of modular reactors and electrolyzers might shift the economic paradigm heavily in favor of integrated nuclear-renewable systems.

This reinforces the value of hybrid multi-resource architectures for sustainable energy researchers: strategies that couple complementary resources to smooth temporal variability, minimize curtailment, and make more efficient use of capital are critical to achieve affordability and reliability on the path to total decarbonization.

The insights from this table serve as the economic reference frame used to conduct the detailed technical performance and sensitivity analyses in the subsequent sections.

### Techno-economic configuration comparison

Table 7 shows some clear techno-economic trade-offs for the different configurations, CapEx denotes the total upfront capital investment cost required for each hybrid configuration, while O&M costs represent annual operating and maintenance expenditures; these annual costs are used consistently in the computation of the ROI over the project lifetime. Both fully renewable Configurations 1 and 2 stand out as having the lowest capital expenditure (CapEx) and fixed O&M (OpEx) costs with ROI periods of 7–10 years. These scenarios also register lower water consumption at 38–40 billion gallons a year, which is advantageous to water stressed island ecosystems like Puerto Rico. Configuration 5, which includes nuclear power, has the highest capital and operational costs but provides the greatest energy reliability, though it also results in the highest water consumption, at 42 billion gallons per year. Hybrid configurations with hydrogen and fuel cells, like configurations 2 and 5, strike a balance between cost, reliability, and resource sustainability. Overall, while renewable-dominant mixes offer short-term economic gains, nuclear-integrated systems may be more favorable for long-term energy security and supply stability.

**Table 7:** Summary of costs, water use, and ROI for energy configurations.

Configuration	Total CapEx (USD)	O&M cost (USD/year)	Water use (gal/year)	ROI (Years)
Geothermal + Solar PV + H_2_ + FC	$75 M	$2 M	38 B	6–13
Wind + Geothermal + H_2_ + FC	$85 M	$5 M	34 B	8–12
Wind + Solar PV + Geothermal + H_2_ + FC	$130 M	$8 M	40 B	10–13
Nuclear + Wind + H_2_ + FC	$150 M	$15 M	41 B	12–35
Nuclear + Wind + Solar PV + H_2_ + FC	$212 M	$26 M	42 B	12–35

### Design trade-offs and implications for hurricane-resilient island grids

Although five hybrid configurations were evaluated, only three were selected as recommended pathways because configurations 2 and 4 exhibited trade-offs that limit their suitability for Puerto Rico’s long-term resilience objectives. Configuration 2 *(Wind + Geothermal + H*$\phantom{0}_2$ *+ FC)* achieves a competitive LCOE (10.4 ¢/kWh) but underperforms in resilience due to its combined reliance on wind and geothermal generation. While geothermal provides firm, weather-independent base-load power, its high upfront capital cost driven by exploratory drilling risk, site-specific resource uncertainty, and limited economies of scale significantly increases system cost when paired with solar PV or wind. In addition, during hurricane events, wind generation becomes highly uncertain, and geothermal capacity alone is insufficient to sustain critical loads over multi-day outages without substantial oversizing of hydrogen storage. This storage oversizing requirement increases both capital expenditure and round-trip efficiency losses, ultimately reducing system robustness. Configuration 4 *(Nuclear + Wind + H*$\phantom{0}_2$ *+ FC)* delivers the lowest LCOE (9.6 ¢/kWh) and strong reliability from firm nuclear base-load support; however, its lower renewability and resilience scores limit its long-term appeal. The absence of solar PV reduces resource diversity and operational flexibility during post-storm recovery, while continued dependence on wind introduces vulnerability under extreme weather conditions. Configuration 5 *(Nuclear + Wind + Solar PV + H*$\phantom{0}_2$ *+ FC)* improves upon this architecture by incorporating solar PV, achieving a more balanced cost–resilience–renewability trade-off with only a marginal LCOE increase.

Beyond Puerto Rico, these findings have important implications for other hurricane-prone Caribbean islands and other island nations with constrained grids, limited land availability, high financing costs, and exposure to prolonged outages. Although the preferred energy sources may differ by island depending on local resource availability, infrastructure, and policy priorities, the general framework developed in this study remains transferable because it is built on dimensionless 3R $\pi$-metrics and a parametric optimization procedure rather than on Puerto Rico-specific absolute scales alone. Puerto Rico therefore serves as a representative case study, while the methodology can be adapted to other island systems with different renewable resource potentials. More broadly, the results suggest that systems combining at least one firm, conventional resource like nuclear micro-reactors and small modular reactors or geothermal with diversified renewables and long-duration storage are generally better positioned to withstand multi-day disruptions than portfolios dominated only by weather-sensitive resources. A further strength of this approach is that it enables efficient preliminary feasibility studies when detailed time-series demand data or long-term weather-resource datasets are limited. In such cases, the parametric optimization framework can still be used to screen technologies, compare hybrid architectures, and identify promising cost-resilience trade-offs before undertaking more data-intensive simulations. This makes the methodology particularly relevant for Caribbean and other small-island settings where data constraints often limit comprehensive evaluation during early planning stages.

The sensitivity analysis reveals important model limitations. In particular, the strong influence of the discount rate indicates that financing conditions can dominate cost-optimal outcomes, with LCOE shifts exceeding 30% under plausible variations. This highlights the critical role of policy instruments such as low-interest financing and loan guarantees.

Again, the use of annualized capacity factors limits the representation of short-term operational dynamics, motivating future extensions toward time-resolved and stochastic modeling framework. Future work could also incorporate demand-side flexibility mechanisms alongside supply-side storage. Recent studies have shown that coordinated aggregation of building thermal inertia can provide substantial flexibility to urban energy systems by shifting heating demand over time, effectively functioning as a form of virtual energy storage^45^. For islanded hybrid systems such as Puerto Rico’s, integrating such building-level thermal flexibility could reduce peak stress, complement hydrogen and battery storage, and potentially lower the physical storage capacity required to satisfy the 3R energy security targets.

### LCOE interpretability using GPR and SHAP

With the aim of deepening understanding of the optimization results, a GPR surrogate model was constructed to capture how the key energy source capacity impacts important outputs like LCOE. The marginal impact of each energy source (Nuclear, Wind, Solar PV, Geothermal, Fuel Cell or BES) on the LCOE is assessed. In this context SHAP (SHapley Additive exPlanations) analysis was performed on the GPR model to evaluate explanatory variables in terms of their contribution toward each output.

Results are presented in Fig. 4, where positive SHAP values indicate an increase in LCOE, while negative values represent cost-reducing contributions. The color scale corresponds to the normalized capacity of each energy source. The analysis shows that higher capacities of fuel cells and wind tend to increase LCOE, whereas greater deployment of nuclear generation and Battery Energy Storage (BES) consistently reduces system cost. This behavior reflects the economic trade-offs between systems relying heavily on storage and intermittent renewables and those supported by stable, high-capacity-factor sources such as nuclear power. The SHAP results from this surrogate model highlights the importance of balancing renewable intermittency with dispatchable base-load generation in achieving economically viable hybrid energy systems.

### Sensitivity analysis of LCOE drivers

Figure 5 illustrates the sensitivity of the LCOE to the techno-economic input parameters used in this work, assisting in identifying the important cost drivers within the energy system model. Each horizontal bar depicts the extent to which LCOE fluctuates with changes of a single input parameter while keeping other factors fixed. Blue segments mark low-impact changes, while red segments reflect high-impact changes.

The distinction is based on the magnitude of LCOE deviation caused by parameter changes: low-impact changes (blue) result in relatively minor deviations from the baseline LCOE, while high-impact changes (red) lead to substantial shifts. The target LCOE of 0.11 USD/kWh is indicated by the vertical dashed line. Critical influencing factors include the discount rate, O&M expenses, fuel costs, and installation costs where LCOE variation is maximized. These factors have a significant impact on the economic viability of energy pathways under uncertain market and regulatory environments.

In contrast, technology efficiency (defined here as the effectiveness of a technology to supply power),

capacity factor, and capital expenditure (CapEx), though still relevant, have lower impact on the LCOE within the range of uncertainty modeled. It is also important to note that shifts in discount rate alone could increase LCOE from under $ 0.10/kWh to more than $ 0.14/kWh , which underscores its prominence and importance for long term financial strategy sensitivity during planning. This assessment provides prioritization lines for Puerto Rico’s policymakers and energy planners, portraying that reduced fixed O&M costs alongside maintaining favorable financing conditions would fundamentally enhance cost-competitiveness. Moreover, the sensitivity analysis highlights Puerto Rico’s need for comprehensive techno-economic modeling to ensure that energy transition pathways remain resilient while aiming for an LCOE of 10 ¢/kWh.

### Recommended hybrid-energy architectures for Puerto Rico

**Fig. 6:**
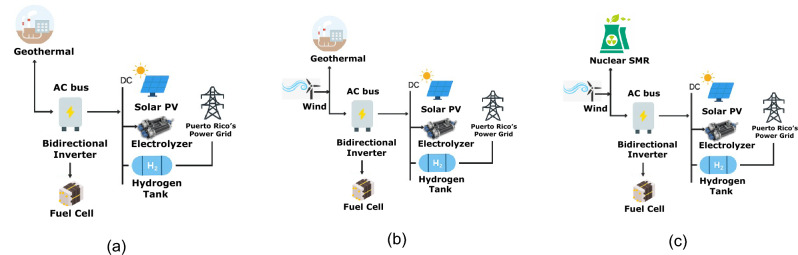
Proposed optimized energy mix for PR: (**a**) Robust energy mix. (**b**) 100 % Renewable energy mix. (**c**) Nuclear-hybrid energy mix.

Figure 6 highlights the three most viable transition pathways for Puerto Rico, which correspond to configurations 1, 3, and 5. As previously shown, these were identified from the optimization process to have the lowest Levelized Cost of Energy (LCOE), feasible energy security metrics, and the highest resilience against outages.

Although each configuration utilizes a different blend of renewable and conventional resources, all of them have an overarching architecture comprising an AC bus connected via a bidirectional inverter to a DC storage loop. The storage loop incorporates batteries with fast cycling capabilities paired with an electrolyzer-H_2_ tank-fuel cell for long-duration backup. The following descriptions detail how these various resource mixes, along with power-electronic designs and storage, minimize cost while maintaining reliability, ease of deployment on the island, and resilience to grid interruptions.

- **Robust Energy Mix** As depicted in Fig. 6a, configuration 1 : *Geothermal + Solar PV + H*_2_ *+ FC*, strengthens Puerto Rico’s pathway to a robust renewable alternative with an optimized LCOE of (12.3 ¢/kWh). In this mix, high penetration solar PV uses Puerto Rico’s strong sunshine to minimize daytime peaks and cut down on fuel imports, while geothermal energy offers a consistent supply that is unaffected by wind lulls or cloudy conditions. Excess daytime generation is routed through the bidirectional inverter into a DC loop where batteries handle sub-hour balancing and surplus electrolytic hydrogen is produced. This hydrogen is then stored on site and may be reconverted by fuel cells during multi-day outages or demand spikes. This scenario cushions the grid against weather-driven variability and hurricane disruptions thanks to the long-term steadiness from geothermal, fast frequency support from batteries, and extended autonomy of hydrogen cells. Since all major assets are modular and commercially viable, phased implementation at community or utility scale is possible, equipping Puerto Rico with disaster-hardy options for high decarbonization.
- **100 % Renewable Energy Mix** In Fig. 6b, configuration 3 :* Wind + Solar PV + Geothermal + H*_2_ *+ FC*, offers Puerto Rico a unique, all-renewable architecture by integrating geothermal, wind and solar PV while also having dual-layer storage. Wind along the coastlines and rooftop or utility-scale solar introduce new weather-diverse inputs that help prevent dips in production simultaneously. Seasonal shifts have little effect on geothermal steady base-load power. Furthermore, any surplus energy from these resources can be stored and used to handle sub-hour ramping within a DC hub, where lithium batteries operate bidirectionally alongside an electrolyzer that converts excess power into hydrogen. The stored H_2_ can be dispatched through a fuel cell after being stored in above ground tanks, allowing for multi-day autonomy when there is prolonged hurricanes that disrupt wind and solar. Coupling geographically spread renewables along with this “battery-for-seconds, hydrogen-for-days” hierarchy strengthens the grid against short pulse disturbances as well as enduring outages without reliance on imported fossil fuels. This all-renewable architecture for Puerto Rico with an optimized LCOE of (11.8 ¢/kWh) integrates scalable modular systems into flexible, resilient utility-scale microgrids, directly supporting the island’s goal of achieving a fully self-sufficient 100% renewable energy grid by 2050.
- **Nuclear-Hybrid Energy Mix** In Fig. 6c, configuration 5 : *Nuclear + Wind + Solar PV + H*_2_ *+ FC*, stands out as the best option since it combines a small modular reactor’s (SMR) steady base-load output with wind and solar’s cost-effective variability, all wrapped within a hydrogen-enabled storage loop permitting multi-day autonomy with an optimized LCOE of (10.0 ¢/kWh). The SMR supplies a stable core output for the station, which reduces the required capacity of batteries and electrolyzers needed to maintain system reliability. The integration of wind turbines and PV arrays further diminishes fuel expenses while decreasing carbon emissions. Surplus renewable energy, together with off-peak nuclear power, drives a DC-linked electrolyzer that fills an on-site H_2_ tank. During prolonged grid outages or evening ramps, high-efficiency fuel cells can reconvert the hydrogen to electricity and preserve critical load supply without spinning reserve overhead. A bidirectional inverter manages the flow of electricity between the AC bus, DC storage chain, and distribution grid, allowing real-time optimization of ramping and ancillary services. Water consumption is flexible, as the actual estimate depends on the kind of small modular reactor chosen. Overall, this configuration offers the lowest LCOE due to the conventional base-load nuclear capacity combined with fast-response batteries alongside season-scale hydrogen storage. This proposed optimal energy mix architecture is the most cost-effective and resilience-focused and energy security-oriented pathway for Puerto Rico’s energy transition.

## Conclusion

This study developed a Puerto Rico–specific optimization framework that integrates techno-economic modeling with dimensionless energy security metrics derived from the Buckingham $\pi$-theorem. The resulting $\pi$-scaling formulation combines economic drivers (LCOE, CAPEX, O&M) with technical variables (generation capacity, storage duration, outage duration, variability, and power limits) to create dimensionless performance indicators applicable across heterogeneous energy resources including SMR, wind, solar PV, geothermal, and hydrogen-based storage systems. By removing units and expressing system behavior through normalized ratios, the framework eliminates size and currency bias, enabling consistent comparison across systems ranging from microgrids to island-scale networks while reducing optimization complexity and clarifying trade-offs between cost, storage coverage, and outage risk.

The proposed 3R metrics are dimensionless, making the framework transferable beyond Puerto Rico to Caribbean islands with differing demand scales, resource availability, and grid maturity. This dimensional consistency ensures scale independence through normalized performance ratios rather than absolute magnitudes, enabling fair techno-economic comparisons across islanded and disaster-prone power systems. Furthermore, the scaling formulation ensures that cost-optimal architectures naturally satisfy minimum resilience thresholds, suggesting that future work could incorporate resilience explicitly within a multi-objective optimization framework. By embedding reliability, resilience, and renewability into the optimization design matrix, the framework moves beyond traditional cost-minimization studies to identify transition pathways that remain both economically viable and hurricane-resilient. This work presents a complementary framework to existing techno-economic planning approaches by combining (i) dimensionless 3R resilience metrics derived from Buckingham $\pi$-theory, (ii) a nonlinear programming formulation capturing economies of scale often simplified in linear energy models, and (iii) Gaussian Process Regression with SHAP-based explainability to quantify uncertainty and attribute cost–resilience trade-offs. Together, these elements create a transparent and scalable methodology linking theoretical modeling with actionable energy planning insights for islanded power systems.

The optimization identified five feasible hybrid energy configurations, from which three representative solutions were proposed to guide Puerto Rico’s 2050 transition goals: the Robust Energy Mix, the 100% Renewable Energy Mix, and the Nuclear–Hybrid Energy Mix. Among these, the nuclear–hybrid configuration achieves the lowest LCOE (10.0 ¢/kWh) while meeting firm-capacity requirements and reducing long-duration storage needs by approximately 20% compared with fully renewable systems. The Robust Energy Mix provides the highest resilience and renewability but at a higher cost of approximately 12.3 ¢/kWh, while the 100% Renewable configuration achieves full renewable penetration at a moderate cost of 11.8 ¢/kWh. Sensitivity analysis further indicates that discount rate, O&M expenditure, and electrolyzer capital costs dominate economic performance, whereas storage sizing most strongly influences resilience outcomes. From a policy-planning perspective, the results indicate that hybrid systems combining firm low-carbon generation with hydrogen buffering can simultaneously satisfy Puerto Rico’s renewable goals while improving system reliability during multi-day outages. Financial mechanisms that reduce electrolyzer and storage capital costs and secure low-interest financing have the greatest impact on delivered electricity price. Among the evaluated options, the Nuclear–Hybrid configuration represents the cost-optimal pathway with reliability of approximately 0.90, resilience of 0.75, and renewability of 0.70, while the Robust and 100% Renewable mixes remain viable alternatives when resilience or renewable penetration are prioritized.

Beyond the Puerto Rico case study, the Buckingham $\pi$-derived 3R parameters establish a transferable dimensional-scaling methodology for evaluating hybrid energy systems across islanded and disaster-prone grids. Unlike conventional techno-economic analyses that remain system-specific, the proposed approach embeds parametric scaling relationships that produce dimensionally consistent and physically grounded energy security metrics, enabling broader application across Caribbean and other hurricane-exposed power systems.

Future work may extend the framework by incorporating geographically resolved renewable resource data, transmission constraints, operational dispatch modeling, and hurricane-damage scenarios. Additional extensions may include spatial optimization of hydrogen infrastructure and integration of supply-chain resilience metrics such as Value-at-Risk (VaR) and Conditional Value-at-Risk (CVaR) for energy commodity logistics^46,47^. These directions would further enhance the robustness of hybrid energy planning under uncertainty and infrastructure disruption.

Ultimately, the dimensionless 3R $\pi$-group formulation introduced in this work establishes a transferable engineering-performance framework that complements optimization-based techno-economic analyses. By enabling consistent evaluation of reliability, resilience, and renewability through normalized metrics, the approach provides a scalable and interpretable decision-support tool for guiding cost-efficient and resilient energy transitions in islanded and disaster-prone power systems facing increasing climate and infrastructure risks.

## Data Availability

The datasets generated during and/or analyzed during the current study are available from the corresponding author upon request.
